# Kidney and uro-trauma: WSES-AAST guidelines

**DOI:** 10.1186/s13017-019-0274-x

**Published:** 2019-12-02

**Authors:** Federico Coccolini, Ernest E. Moore, Yoram Kluger, Walter Biffl, Ari Leppaniemi, Yosuke Matsumura, Fernando Kim, Andrew B. Peitzman, Gustavo P. Fraga, Massimo Sartelli, Luca Ansaloni, Goran Augustin, Andrew Kirkpatrick, Fikri Abu-Zidan, Imitiaz Wani, Dieter Weber, Emmanouil Pikoulis, Martha Larrea, Catherine Arvieux, Vassil Manchev, Viktor Reva, Raul Coimbra, Vladimir Khokha, Alain Chichom Mefire, Carlos Ordonez, Massimo Chiarugi, Fernando Machado, Boris Sakakushev, Junichi Matsumoto, Ron Maier, Isidoro di Carlo, Fausto Catena, WSES-AAST Expert Panel, Paola Fugazzola, Martijn Stommel, Mohan Rajashekar, Edward Tan, Matti Tolonen, Marco Ceresoli, Carlos Augusto Gomez, Niccolo Allievi, Mircea Chirica, Francesco Salvetti, Riccardo Bertelli, Offir Ben-Ishay, Hany Bahouth, Gianluca Baiocchi, Antonio Tarasconi, Stefania Cimbanassi, Osvaldo Chiara, Richard ten-Broek, Giulia Montori, Erika Picariello, Leonardo Solaini, Andreas Hecker, Matteo Tomasoni, Paola Perfetti, Neil Parry, Nicola DeAngelis, Bruno M. Pereira, Joaquin Bado, Oreste Romeo, Andreas Pikoulis, Miklosh Bala, Lena Napolitano, Joseph Galante, Sandro Rizoli, Paula Ferrada, Tal Horer, Megan Brenner, Rao Ivatury

**Affiliations:** 1 General, Emergency and Trauma Surgery, Pisa University Hospital, Via Paradisia, 56124 Pisa, Italy; 2 Trauma Surgery, Denver Health, Denver, CO USA; 3 Division of General Surgery Rambam Health Care Campus, Haifa, Israel; 4 Trauma Surgery Dept., Scripps Memorial Hospital, La Jolla, California USA; 5 General Surgery Dept., Mehilati Hospital, Helsinki, Finland; 6 Department of Emergency and Critical Care Medicine, Chiba University Hospital, Chiba, Japan; 7 Urology Department, University of Colorado, Denver, USA; 8 Surgery Department, University of Pittsburgh, Pittsburgh, PA USA; 9 Trauma/Acute Care Surgery & Surgical Critical Care, University of Campinas, Campinas, Brazil; 10 General and Emergency Surgery, Macerata Hospital, Macerata, Italy; 11 General, Emergency and Trauma Surgery Department, Bufalini Hospital, Cesena, Italy; 12 Department of Surgery, Zagreb University Hospital Centre and School of Medicine, University of Zagreb, Zagreb, Croatia; 13 General, Acute Care, Abdominal Wall Reconstruction, and Trauma Surgery, Foothills Medical Centre, Calgary, Alberta Canada; 14 Department of Surgery, College of Medicine and Health Sciences, UAE University, Al-Ain, United Arab Emirates; 15 Department of Surgery, DHS Hospitals, Srinagar, Kashmir India; 16 Department of General Surgery, Royal Perth Hospital, Perth, Australia; 17 3rd Department of Surgery, Attiko Hospital, National & Kapodistrian University of Athens, Athens, Greece; 18 General Surgery, “General Calixto García”, Habana Medicine University, Havana, Cuba; 19 Clin. Univ. de Chirurgie Digestive et de l’Urgence, CHUGA-CHU Grenoble Alpes UGA-Université Grenoble Alpes, Grenoble, France; 20 General and Trauma Surgery Department, Pietermaritzburg Hospital, Pietermaritzburg, South Africa; 21 General and Emergency Surgery, Sergei Kirov Military Academy, Saint Petersburg, Russia; 22 Department of General Surgery, Riverside University Health System Medical Center, Moreno Valley, CA USA; 23 General Surgery Department, Mozir City Hospital, Mozir, Belarus; 24 Department of Surgery and Obstetrics and Gynecology, University of Buea, Buea, Cameroon; 25 Trauma and Acute Care Surgery, Fundacion Valle del Lili, Cali, Colombia; 26 General and Emergency Surgery Department, Montevideo Hospital, Montevideo, Paraguay; 27 General Surgery Department, Medical University, University Hospital St George, Plovdiv, Bulgaria; 28 Department of Emergency and Critical Care Medicine, Saint-Marianna University School of Medicine, Kawasaki, Japan; 29 Department of Surgery, Harborview Medical Centre, Seattle, USA; 30 Department of Surgical Sciences and Advanced Technologies “GF Ingrassia”, Cannizzaro Hospital, University of Catania, Catania, Italy; 31 Emergency and Trauma Surgery, Maggiore Hospital, Parma, Italy

**Keywords:** Kidney, Urogenital, Urethra, Ureter, Bladder, Trauma, Adult, Pediatric, Classification, Guidelines, Embolization, Surgery, Operative, Non-operative, Conservative, Stenting, Urological, Endovascular trauma management, Flow chart

## Abstract

Renal and urogenital injuries occur in approximately 10-20% of abdominal trauma in adults and children. Optimal management should take into consideration the anatomic injury, the hemodynamic status, and the associated injuries. The management of urogenital trauma aims to restore homeostasis and normal physiology especially in pediatric patients where non-operative management is considered the gold standard. As with all traumatic conditions, the management of urogenital trauma should be multidisciplinary including urologists, interventional radiologists, and trauma surgeons, as well as emergency and ICU physicians. The aim of this paper is to present the World Society of Emergency Surgery (WSES) and the American Association for the Surgery of Trauma (AAST) kidney and urogenital trauma management guidelines.

## Background

In both, adult and children cohorts, urogenital trauma has a cumulative incidence of 10-20%, and the kidney is involved in 65–90% of the time [1–3]. Males are involved 3 times more than females (both in adults and children) [2, 4]. As in other abdominal injuries, the use of non-operative management (NOM) has significantly increased in last decades, particularly due to the introduction of hybrid rooms and endovascular trauma and bleeding management (EVTM) associated with modern urological mini-invasive procedures [5, 6]. Moreover, In pediatric patients, NOM should be the first option as soon as it is viable and safe. However, operative management (OM) remains the gold standard in unstable patients, after failure of NOM (fNOM), and in many injuries caused by penetrating mechanisms; in fact, in gunshot and stab wounds, OM is applied in 75% and 50% of cases, respectively [1]. As for the other abdominopelvic lesion management, decisions should be based on physiology, anatomy, and associated injuries [6–9]. Another important consideration relates to the different management approach to kidney and urological trauma urologists and trauma surgeons [10]. Urologic guidelines tend in general to focus more on organ preservation, whereas trauma surgeons tend to consider the stabilization of physiology more importantly than organ preservation [10]. Despite this different point of view, an integrated approach and active collaboration between the two specialties forms the basis to achieve optimal management and the best outcomes [10]. This is particularly true for urogenital and urinary tract injuries in which the multidisciplinary approach is the cornerstone to improve short- and long-term outcomes.

## Notes on the use of the guidelines

The guidelines are evidence-based, with the grade of recommendation based on the evidence. The guidelines present the diagnostic and therapeutic methods for optimal management of urogenital trauma. The practice guidelines promulgated in this work do not represent a standard of practice. They are suggested plans of care, based on the best available evidence and the consensus of experts, but they do not exclude other approaches as being within the standard of practice. For example, they should not be used to compel adherence to a given method of medical management, which method should be finally determined after taking account of the conditions at the relevant medical institution (staff levels, experience, equipment, etc.) and the characteristics of the individual patient. However, responsibility for the results of treatment rests with those who are directly engaged therein, and not with the consensus group.

## Methods

**Fig. 1:**
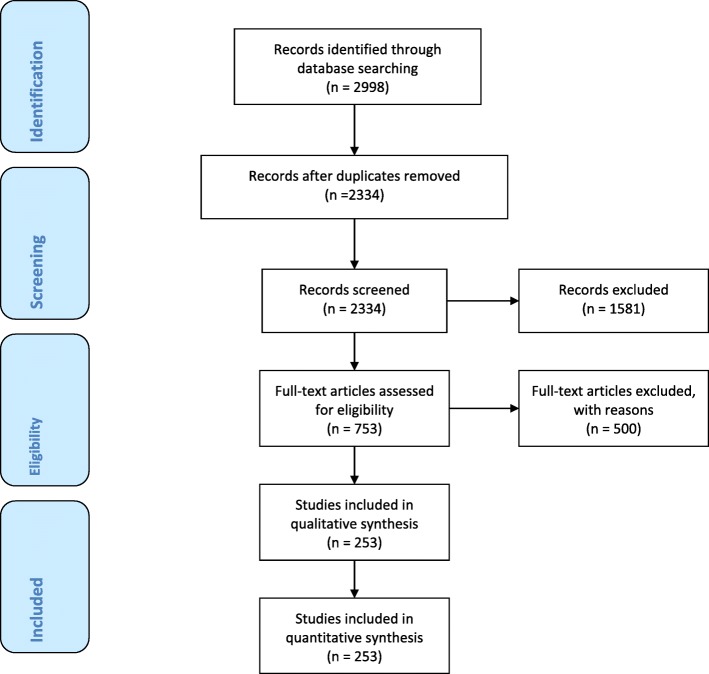
PRISMA flow chart

A computerized search was done by the bibliographer in different databanks (MEDLINE, Scopus, EMBASE) and citations were included for the period between January 1990 and August 2018 using the primary search strategy: kidney, injuries, trauma, urogenital, adult, pediatric, hemodynamic instability/stability, angioembolization, management, nonoperative, conservative, operative, surgery, diagnosis, follow-up, combined with AND/OR. No search restrictions were imposed. The dates were selected to allow comprehensive published abstracts of clinical trials, consensus conference, comparative studies, congresses, guidelines, government publication, multicenter studies, systematic reviews, meta-analysis, large case series, original articles, and randomized controlled trials. Case reports and small case series were excluded. Narrative review articles were also analyzed to determine if other cited studies should be included. The literature selection is reported in the flow chart (Fig. 1).

The level of evidence (LE) was evaluated using the GRADE system [11] (Table 1).

**Table 1:** GRADE system to evaluate the level of evidence and recommendation

Grade of recommendation	Clarity of risk/benefit	Quality of supporting evidence	Implications
1A			
Strong recommendation, high-quality evidence	Benefits clearly outweigh risk and burdens, or vice versa	RCTs without important limitations or overwhelming evidence from observational studies	Strong recommendation, applies to most patients in most circumstances without reservation
1B			
Strong recommendation, moderate-quality evidence	Benefits clearly outweigh risk and burdens, or vice versa	RCTs with important limitations (inconsistent results, methodological flaws, indirect analyses or imprecise conclusions) or exceptionally strong evidence from observational studies	Strong recommendation, applies to most patients in most circumstances without reservation
1C			
Strong recommendation, low-quality or very low-quality evidence	Benefits clearly outweigh risk and burdens, or vice versa	Observational studies or case series	Strong recommendation but subject to change when higher quality evidence becomes available
2A			
Weak recommendation, high-quality evidence	Benefits closely balanced with risks and burden	RCTs without important limitations or overwhelming evidence from observational studies	Weak recommendation, best action may differ depending on the patient, treatment circumstances, or social values
2B			
Weak recommendation, moderate-quality evidence	Benefits closely balanced with risks and burden	RCTs with important limitations (inconsistent results, methodological flaws, indirect or imprecise) or exceptionally strong evidence from observational studies	Weak recommendation, best action may differ depending on the patient, treatment circumstances, or social values
2C			
Weak recommendation, Low-quality or very low-quality evidence	Uncertainty in the estimates of benefits, risks, and burden; benefits, risk, and burden may be closely balanced	Observational studies or case series	Very weak recommendation; alternative treatments may be equally reasonable and merit consideration

A group of experts in the field coordinated by a central coordinator was contacted to express their evidence-based opinion on several issues about the pediatric (< 16 years old) and adult urogenital trauma [12, 13]. Urogenital trauma was assessed by the anatomy of the injury (kidney, urogenital tract, bladder), type of injury (blunt and penetrating injury), management (conservative and operative management), and type of patient (adults, pediatrics). Through the Delphi process, different issues were discussed in subsequent rounds. The central coordinator assembled the different answers derived from each round. Each version was then revised and improved. The definitive version was discussed during the WSES World Congress (in June 2019 in Njimengen, The Netherlands) by a combined expert group from both societies (WSES-AAST). The final version about which the agreement was reached resulted in the present manuscript. Statements are summarized in Table 3.

### Definitions

*In adult patients, hemodynamic instability* is considered the condition in which admission systolic blood pressure upon admission is < 90 mmHg with evidence of skin vasoconstriction (cool, clammy, decreased capillary refill), altered level of consciousness and/or shortness of breath, or > 90 mmHg but requiring bolus infusions/transfusions and/or vasopressor drugs and/or admission base excess (BE) > − 5 mmol/l and/or shock index > 1 and/or transfusion requirement of at least 4–6 Units of packed red blood cells within the first 24 h. *Transient responder patients* (adult and pediatric) are those showing an initial response to adequate fluid resuscitation, but then subsequent signs of ongoing blood loss and perfusion deficits. These patients have an initial response to therapy but do not reach sufficient stabilization to undergo interventional radiology procedures or NOM.

*In pediatric patients, hemodynamic stability* is considered a systolic blood pressure of 90 mmHg plus twice the child’s age in years (the lower limit is inferior to 70 mmHg plus twice the child’s age in years, or inferior to 50 mmHg in some studies). An acceptable hemodynamic status in children is considered a positive response to fluid resuscitation: 3 boluses of 20 mL/kg of crystalloid replacement should be administered before blood replacement leading to heart rate reduction, cleared sensorium, return of peripheral pulses, normal skin color, increase in blood pressure and urinary output, and an increase in warmth of the skin in the extremities. Clinical judgment however is fundamental in evaluating children.

### WSES classification

**Fig. 2:**
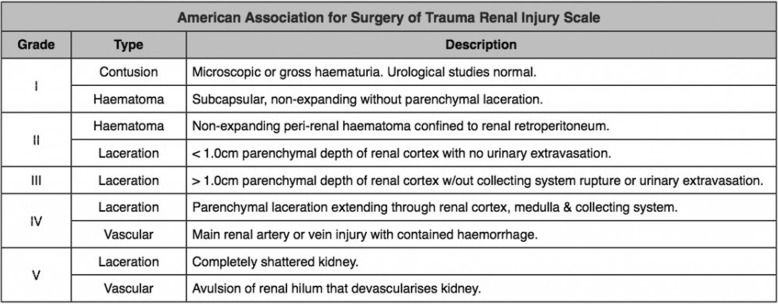
AAST organ injury scale for kidney trauma

The WSES Classification (Table 2) divides kidney injuries into four classes considering the AAST-OIS classification (Fig. 2) and the hemodynamic status (Table 3):

- **Minor** (WSES class I)
- **Moderate** (WSES class II)
- **Severe** (WSES class III and IV)

**Table 2:** WSES kidney trauma classification

	WSES grade	AAST	Hemodynamic
Minor	WSES grade I	I–II	Stable
Moderate	WSES grade II	III or segmental vascular injuries	Stable
Severe	WSES grade III	IV–V or any grade parenchymal lesion with main vessels dissection/occlusion	Stable
	WSES grade IV	Any	Unstable

**Table 3:** Statements summary

Statements	
Diagnostic procedures	
• Kidney	- The choice of diagnostic method upon admission depends on the hemodynamic status of the patient. (GoR 1A) - E-FAST is effective and rapid to detect intra-abdominal free fluid. (GoR 1A) - E-FAST has low sensitivity and specificity in kidney trauma. (GoR 1B) - Contrast-enhanced CT scan associated with delayed urographic phase is the gold standard in hemodynamic stable or stabilized adults after blunt of penetrating trauma and in severely injured children when kidney or urinary tract injury is suspected. (GoR 1A) - In blunt trauma, contrast-enhanced CT scan associated with delayed urographic phase must be performed in cases of macro- or micro- hematuria with hypotension and after high-energy deceleration trauma regardless of the presence of hematuria. (GoR 2B) - In penetrating trauma, contrast-enhanced CT scan associated with delayed urographic phase is indicated in all hemodynamic stable or stabilized patients. (GoR 1B) - Pediatric patients with high energy/penetrating/decelerating trauma and/or in cases of drop in hematocrit associated with any degree of hematuria should undergo contrast-enhanced CT-scan with delayed urographic phase. (GoR 2A) - Ultrasound, contrast-enhanced US and eco-Doppler (E-FAST excluded) are generally not recommended as diagnostic tools during the initial evaluation of adult patients with high-energy trauma when multiple injuries and/or injury to the urinary tract and collecting system are suspected. (GoR 1C) - Ultrasound, contrast-enhanced US, and eco-doppler can be used in pregnant women and in the pediatric population as an alternative to CT-scan in the presence of hemodynamic stability during the immediate assessment and in follow-up evaluations. (GoR 1C) - In children with mild symptoms, minimal clinical findings, hematuria <50 RBCs/HPF and no other indications of CT-scanning, ultrasound and/or contrast-enhanced US and/or eco-doppler associated to blood test may be adopted for the initial evaluation. (GoR 2A) - Intravenous urography may be useful in unstable patients during surgery when a kidney injury is found intraoperatively or when CT-scanning is not available and a urinary tract injury is suspected. (GoR 2C*)*
• Ureter	- Injury to the ureter should be suspected in high-energy blunt trauma, particularly in deceleration injuries with multi-system involvement and in all penetrating abdominal trauma. (GoR 1C). - Intravenous contrast-enhanced CT-scan with delayed phase should be performed in hemodynamically stable or stabilized patients if ureteral injury is suspected (GoR 1C) - Direct inspection of the ureter should be always performed during emergency laparotomy in patients with suspected ureteral injury. (GoR 1C)
• Bladder	- Retrograde cystography (conventional radiography or CT-scan) represents the diagnostic procedure of choice in bladder injuries. (GoR 1C) - Retrograde cystography should be always performed in hemodynamically stable or stabilized patients with suspected bladder injury. (GoR 1C) - Intravenous contrast-enhanced CT-scan with delayed phase is less sensitive and specific than retrograde cystography in detecting bladder injuries. (GoR 1B) - In pelvic bleeding amenable to angioembolization associated to suspected bladder injuries, cystography should be postponed until the completion of the angiographic procedure to avoid affecting the accuracy of angiography. (GoR 2A) - Direct inspection of the intraperitoneal bladder, whenever feasible, should always be performed during emergency laparotomy in patients with suspected bladder injury. Methylene blue or indigo carmine could be useful in intraoperative investigation. (GoR 1C)
• Urethra	- Patients with post-traumatic urethral hemorrhage should be investigated for urethral injuries. (GoR 1C) - During emergency laparotomy, if an urethral injury is suspected, it should be investigated directly whenever feasible. (GoR 2A) - Retrograde urethrography and selective urethroscopy represent the modalities of choice to investigate traumatic urethral injuries. (GoR 1B) - In the event of penile lesions, urethroscopy should be preferred to retrograde uretrography (GoR 2A)
Management	
Kidney Non-operative management (NOM)	- NOM should be the treatment of choice for all hemodynamical stable or stabilized minor (AAST I-II), moderate (AAST III) and severe (AAST IV-V) lesions. (GoR 1B) - Only in selected settings, with immediate availability of operating room, surgeons and adequate resuscitation, immediate access to blood, blood products and to high dependency / intensive care environment, and without other reasons for surgical exploration, NOM may be considered even in hemodynamically transient responder patients. (GoR 2C) - In deciding for NOM in hemodynamically stable or stabilized patients, accurate classification of the degree of injury and associated injuries with CT-scan with intravenous contrast and delayed urographic phases is mandatory. (GoR 2A) - NOM in penetrating lateral kidney injuries is feasible and effective but accurate patient selection is crucial even in the absence of other indications for laparotomy. In particular, cases without violation of the peritoneal cavity are more suitable for NOM. (GoR 2A) - Isolated urinary extravasation, in itself, is not an absolute contra-indication to NOM in absence of other indications for laparotomy. (GoR 1B) - In low resource settings, NOM could be considered in hemodynamically stable patients without evidence of associated injuries, with negative serial physical examinations and negative first level imaging and blood tests. (GoR 2C)
Kidney Angiography and angioembolization	- Angiography with eventual super-selective angioembolization is a safe and effective procedure; it may be indicated in hemodynamically stable or stabilized patients with arterial contrast extravasation, pseudoaneurysms, arteriovenous fistula, and non-self-limiting gross hematuria. (GoR 1C) - Angioembolization should be performed as selectively as possible. (GoR 1C) - Blind-angioembolization is not indicated in hemodynamically stable or stabilized patients with both kidneys when angiography is negative for active bleeding, regardless of arterial contrast extravasation on CT-scan. (GoR 1C) - In hemodynamically stable or stabilized patients with severe renal trauma with main renal artery injury, dissection or occlusion, angioembolization and/or percutaneous revascularization with stent or stentgraft is indicated in specialized centres and in patients with limited warm ischemia time (<240 min) (GoR 2C) - Endovascular selective balloon occlusion of the renal artery could be utilized as a bridge to definitive hemostasis. This procedure requires direct visualization by fluoroscopy where the balloon is advanced over a selectively placed guidewire. (GoR 2B) - In severe injury with main renal vein injury without self-limiting bleeding, angioembolization is not indicated. Patients should undergo surgical intervention. (GoR 1C) - In hemodynamically stable or stabilized patients with solitary kidney and moderate (AAST III) or severe (AAST IV-V) renal trauma with arterial contrast extravasation on CT-scan, angiography with eventual super-selective angioembolization should be considered as the first choice. (GoR 1C) - In hemodynamically stable or stabilized patients with active kidney bleeding at angiography and without other indications for surgical intervention, in case of failure of the initial angioembolization, a repeat angioembolization should be considered. (GoR 1C) - In adults, only in selected setting (immediate availability of operating room, surgeon, adequate resuscitation, immediate access to blood and blood products and to high dependency / intensive care environment) and without other reasons for surgical exploration, angioembolization might be considered in selected hemodynamically transient responder patients. (GoR 2C) - In children, angiography and eventual super-selective angioembolization should be the first choice even with active bleeding and labile hemodynamics, iof there is immediate availability of angiographic suite, immediate access to surgery and to blood and blood products, and to high dependency / intensive care environment. (GoR 2C)
Kidney Operative management (OM)	- Hemodynamically unstable and non-responder (WSES IV) patients should undergo OM. (GoR 2A) - Resuscitative Endovascular Balloon Occlusion of the Aorta (i.e., REBOA) may be used in hemodynamically unstable patients as a bridge to other more definitive procedures for hemorrhage control. (GoR 2B) - In cases of severe renal vascular injuries without self-limiting bleeding, OM is indicated. (GoR 1C) - The presence of non-viable tissue (devascularized kidney) is not an indication to OM in the acute setting in the absence of other indications for laparotomy. (GoR 2A) - Hemodynamic stable or stabilized patients having damage to the renal pelvis not amenable to endoscopic/percutaeous techniques/stent should be considered for delayed OM in absence of other indications for immediate laparotomy. (GoR 2B)
Urinary tract injuries	
• Ureter	- Contusions may require ureteral stenting when urine flow is impaired. (GoR 1C) - Partial lesions of the ureter should be initially treated conservatively with the use of a stent, with or without a diverting nephrostomy in the absence of other indications for laparotomy. (GoR 1C) - Partial and complete ureteral transections or avulsion not suitable for NOM may be treated with primary repair plus a double J stent or ureteral re-implant into the bladder in case of distal lesions (GoR 1C). - Ureteral injuries should be repaired operatively when discovered during laparotomy or in cases where conservative management has failed (GoR 1C) - Ureteral stenting should be attempted in cases of partial ureteral injuries diagnosed in a delayed fashion; if this approach fails, and/or in case of complete transection of the ureter, percutaneous nephrostomy with delayed surgical repair is indicated. (GoR 1C) - In any ureteral repair, stent placement is strongly recommended. (GoR 1C)
• Bladder	- Bladder contusion requires no specific treatment and might be observed clinically. (GoR 1C) - Intraperitoneal bladder rupture should be managed by surgical exploration and primary repair (GoR 1B) - Laparoscopy might be considered in repairing isolated intraperitoneal injuries in case of hemodynamic stability and no other indications for laparotomy. (GoR 2B) - In case of severe intraperitoneal bladder rupture, during damage control procedures, urinary diversion via bladder and perivesical drainage or external ureteral stenting may be used. (GoR 1C) - Uncomplicated blunt or penetrating extraperitoneal bladder injuries may be managed non-operatively, with urinary drainage via a urethral or suprapubic catheter in the absence of other indication for laparotomy. (GoR 1C) - Complex extra-peritoneal bladder ruptures—i.e., bladder neck injuries, lesions associated to pelvic ring fracture and/or vaginal or rectal injuries- should be explored and repaired. (GoR 1C) - Surgical repair of extraperitoneal bladder rupture should be considered during laparotomy for other indications and during surgical exploration of the prevesical space for orthopedic fixations. (GoR 1C) - In adult patients, urinary drainage with urethral catheter (without suprapubic catheter) after surgical management of bladder injuries is mandatory (GoR 1B); for pediatric patients suprapubic cystostomy is recommended (GoR 2C)
• Urethra	- Urinary drainage should be obtained as soon as possible in case of traumatic urethral injury. (GoR 1C) - Blunt anterior urethral injuries should be initially managed conservatively with urinary drainage (via urethral or suprapubic catheter); endoscopic treatment with realignment should be attempted before surgery. Delayed surgical repair should be considered in case of failure of conservative treatment after endoscopic approach. (GoR 1C) - Partial blunt injuries of the posterior urethra may be initially managed conservatively with urinary drainage (via urethral or suprapubic catheter) and endoscopic realignment; definitive surgical management should be delayed for 14 days if no other indications for laparotomy exist. (GoR 1C) - Injuries of the posterior urethra in cases of hemodynamic instability should be approached by immediate urinary drainage and delayed treatment. (GoR 1C) - Conservative treatment of penetrating urethral injuries is generally not recommended. (GoR 1C) - Penetrating injuries of anterior urethra should be treated with immediate direct surgical repair if the clinical conditions allow and if an experienced surgeon is available; otherwise, urinary drainage should be performed and delayed treatment planned. (GoR 1C) - Penetrating injuries of the posterior urethra should be treated with primary repair only if the clinical conditions allow. Otherwise, urinary drainage and delayed urethroplasty is recommended. (GoR 1C) - When posterior urethral injury is associated with complex pelvic fracture, definitive surgical treatment with urethroplasty should be performed after the healing of pelvic ring injury. (GoR 1C*)*
Short- and long-term follow-up	
Kidney and urinary tract	- Follow-up imaging is not required for minor (AAST I-II) renal injuries managed non-operatively. (GoR 2B) - In moderate (AAST III) and severe (AAST IV-V) renal injuries, the need for follow-up imaging is driven by the patients’ clinical conditions. (GoR 2B) - In severe injuries (AAST IV-V), contrast-enhanced CT scan with excretory phase (in cases with possible or documented urinary extravasation) or ultrasound and contrast-enhanced US are suggested within the first 48 h after trauma in adult patients and in delayed follow-up. (GoR 2A) - Follow-up imaging in pediatric patients should be limited to moderate (AAST III) and severe (AAST IV-V) injuries. (GoR 2B) - In pediatric patients, ultrasound and contrast-enhanced US should be the first choice in the early and delayed follow-up phases. If cross-sectional imaging is required, magnetic resonance should be preferred. (GoR 2B) - CT-scan with delayed phase imaging is the method of choice for the follow-up of ureteral and bladder injuries. (GoR 2A) - Uretroscopy or uretrogram are the methods of choice for the follow-up of urethral injuries. (GoR 2A) - Return to sport activities should be allowed only after microscopic hematuria is resolved. (GoR 2B)

***Minor kidney injuries:***

- ***WSES class I*** includes hemodynamically stable AAST-OIS grade I–II blunt and penetrating lesions.

***Moderate kidney injuries:***

- ***WSES class II*** includes hemodynamically stable AAST-OIS grade III blunt and penetrating lesions.

***Severe kidney injuries:***

- ***WSES class III*** includes hemodynamically stable AAST-OIS grade IV–V blunt and penetrating lesions and any grade parenchymal lesion with arterial dissection/occlusion.
- ***WSES class IV*** includes hemodynamically unstable AAST-OIS grade I–V blunt and penetrating lesions

**Fig. 3:**
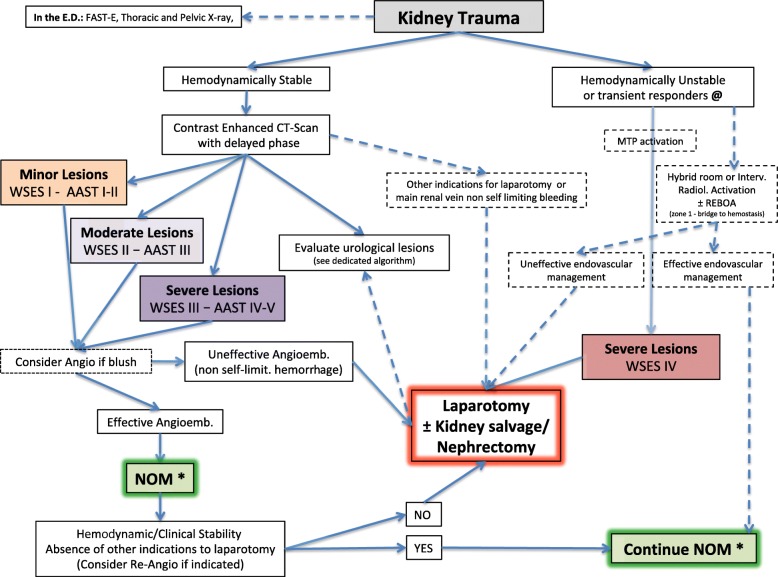
Kidney trauma management algorithm

**Fig. 4:**
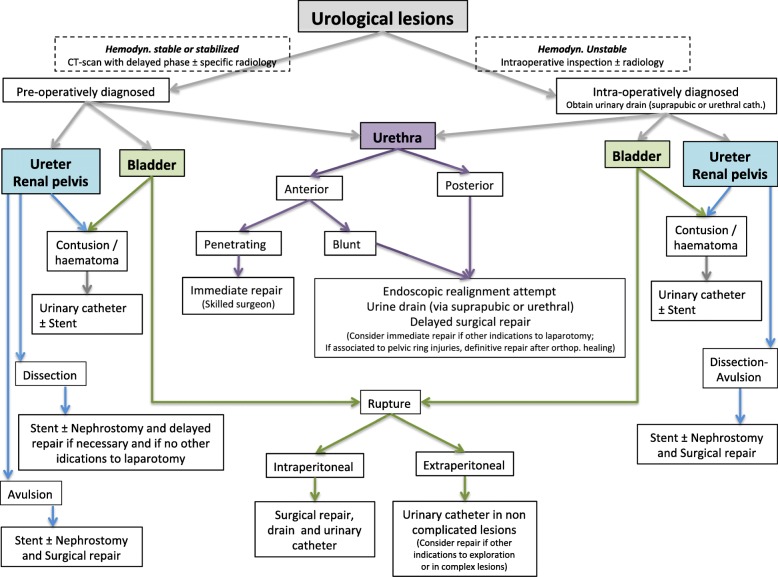
Uro-trauma management algorithm

Based on the present classification, WSES and AAST suggest a management algorithm for kidney injury shown in Fig. 3 and for urogenital tract injuries in Fig. 4.

### Patient stratification

During the initial evaluation the hemodynamic status, mechanism of injury, presence of associated injuries, and anamnestic data must be considered (i.e., previous renal injuries, previous renal surgery, congenital single or pathologic kidneys or diseases), especially in children.

In **adults**, the clinical examination in urogenital trauma should consider the presence of hematuria, flank/abdominal pain/contusion, rib fractures, and mechanism of trauma. Special attention should be given to pelvic trauma in which urethral injuries can be frequently missed but should ideally be diagnosed in the first hours [1]. Macro or micro-hematuria is frequently present (88-94%) in cases of renal/urogenital trauma but it does not predict the grade of injury [13, 14]. Macro-hematuria is more frequently associated with major renal injuries; however, in 10–25% of high-grade kidney injury hematuria is, the same being observed in 24–50% of ureteropelvic junction and renal hilum injuries [13, 15]. In 0.1–0.5% of the patients, hemodynamic stability and micro-hematuria exist in the presence of a significant urinary tract injury [5, 16–18].

In **children,** the kidney is commonly injured following blunt trauma because of many anatomical reasons: less perirenal fat, thinner abdominal muscles, lack of ossification of the rib cage, larger kidney size, and fetal kidney lobulations, making them more vulnerable to injury [2, 3, 19–23]. However, even in the pediatric population, there is no clear correlation between the presence and type of hematuria and the degree of kidney injury (36–40% of renal injuries and in up to 24% of renal artery occlusions hematuria is absent) [22, 24]. However, while micro-hematuria (< 50 red blood cells (RBC) per high-power field (HPF)) is frequent in children due to the kidney anatomy and the presence of undiagnosed kidney diseases (1–36%), macro-hematuria seems to be more related to major renal injuries [22, 24]. The general suggestion is to perform imaging investigation in all those patients with blunt trauma with > 50 RBCs/HPF [13, 22]. In order to refine the use of CT scan in children, however, other factors should be considered (i.e., mechanism of injury and its energy/degree of deceleration associated with physical findings such as hypotension, flank hematoma and ecchymosis, rib fractures, cutaneous signs in the abdomen, and a drop in hematocrit associated with any degree of hematuria) [3, 13, 14, 17, 19–22, 24–34]. On the other hand, in children with minimal symptoms and/or clinical findings and < 50 RBCs/HPF, ultrasound (US), contrast-enhanced ultrasound (CEUS), Eco-Doppler, and clinical and blood test monitoring may be sufficient for the initial evaluation [22].

In penetrating injuries, the presence of hematuria does not correlate with the grade of kidney injury. However, penetrating injuries are commonly associated with other intra-abdominal injuries [18, 26, 31, 35], therefore, independently from the degree of hematuria, all hemodynamically stable patients should be imaged following a penetrating mechanism of injury [18].

### Pathophysiology of injury

#### Kidney

The most common mechanism of injury involving the kidney is blunt trauma associated frequently to high-velocity deceleration (90% of cases); whereas penetrating trauma (gunshot and stab wounds occur in 1.4–3.3% [5, 16–18, 36]. However, these incidences depend on the geographic area of the world [37].

The kidney is well protected in the retroperitoneum; however, it is particularly vulnerable to blunt trauma accompanied by rapid deceleration because the kidney is fixed only by the renal pelvis in the uretero-pelvic junction and by the vascular pedicle. In **adults,** the most frequent blunt mechanisms are falls from height, assault, skiing accidents, and road traffic–related injuries. In **children,** sports injuries such as skiing, snowboarding, horse riding, and bicycle and motorcycle accidents are the most frequent [2, 3, 14, 21, 23, 38–40]. In the pediatric population, isolated blunt injuries are more frequent and occur after 5 years of age, while penetrating injuries usually increase after 14 years of age [2, 4]. Penetrating trauma can affect the kidneys especially when the superior abdomen is involved [5]. Isolated penetrating kidney injuries are rare and renal vascular injuries are more frequent than in blunt trauma [1, 22]. The majority of renal injuries (up to 90%) are minor both in adults and children and involve the parenchyma or segmental vessels [2, 16]. A unique and uncommon type of injury is the isolated renal arterial transection or intimal disruption which occurs particularly in cases of rapid deceleration [1].

#### Ureter

Traumatic ureteral lesions are rare (less than 1%) [41, 42]. The most common cause of ureteral injury is penetrating trauma, especially gunshot wounds [43–46]; only 1/3 of cases are caused by blunt trauma [47]. As opposed to stab wounds, gunshot wounds can produce a blast effect even at a distance of 2 cm from the bullet path [41, 48]. In blunt trauma, ureteral injuries commonly happen at the ureteropelvic junction, especially in children and in high energy deceleration injuries [41, 44, 45, 48, 49]. Associated organ injuries are common in case of ureteral lesions [42, 45, 50]. The clinical presentation of ureteral injuries might be subtle but isolated hematuria is a common finding.

#### Bladder

Bladder injury is more frequent following blunt than penetrating trauma (65–86% vs. 14–35%) [51–53]. In particular, bladder injury is present in 3.6% of abdominal gunshot injuries and 20% of penetrating buttock injuries [41, 48, 54]. Due to the high energy necessary to damage the bladder, 60 to 90% of patients presenting with bladder injury have a pelvic bony fracture while 6–8% of patients with a pelvic fracture will have bladder injury [41, 48, 49, 54]. Pediatric patients are more susceptible to bladder injuries due to the children anatomy. However, bladder injuries in children are less associated with pelvic fractures than in adults [55]. A Pelvic fracture with hematuria is associated to a bladder injury in 30% of cases [45, 49]. Associated prostate-urethral injuries and rupture of the bladder occur in 10–29% male patients [45].

Bladder injuries are mainly of four types: intra-peritoneal bladder rupture (IBR), extra-peritoneal bladder rupture (EBR), bladder contusion and bladder neck avulsion. IBR occurs in 15–25% of cases [41, 45, 48, 49]. EBR is the most common and is found in 60–90% of patients, and it is more frequently associated with pelvic fractures [48]. Combined Bladder Rupture (CBR), i.e., a combination of IBR and EBR, is found in 5–12% of cases [41, 48, 56]. EBR can be further classified into simple EBR, where the urinary leak is limited to the extra-peritoneal pelvic region, and complex injuries where extravasated urine infiltrates the anterior abdominal wall, the scrotum, and the perineum [48].

#### Urethra

Urethral injuries are uncommon; they mostly affect male patients and are usually diagnosed following blunt trauma [45, 57]. Urethral injuries are divided into anterior (bulbar and penile urethra) and posterior injuries (proximal to the perineal membrane, at the prostatic or membranous urethra). The main cause of anterior urethral injury is direct blunt trauma [45, 48, 50]. Penetrating injuries to the anterior urethra are rare and are mainly caused by gunshot injuries [58, 59].

Injuries to the posterior urethra usually result from pelvic trauma, Pelvic fracture urethral injury (PFUI), present in 1.5–5% of anterior pelvic fractures [60, 61]. The risk of urethral injury increases by 10% for every 1-mm increase in pubic symphysis diastasis [62]. Posterior urethral injuries may be classified as complete (65% of lesions) or incomplete (35% of cases) [63]. In complete injuries, a gap is present between the two injured stumps of the urethra. Penetrating injuries to the posterior urethra are extremely rare and are caused mainly by gunshot wounds; the risk of associated intra-abdominal lesions is high [64]. The Goldman classification of urethral injuries [65] includes five types of lesions aimed at discerning anterior from posterior and complete from incomplete and at determining whether posterior urethral injuries involve the bladder neck or the rectal wall. Associated urethral and bladder injuries are found in up to 20% of cases [66]. Female urethral injuries are uncommon and are often caused by pelvic injuries and are usually associated with rectal and vaginal injuries [67, 68].

### Diagnostic procedures

There are no specific recommendations regarding the diagnosis of urogenital injuries in children. Therefore, pediatric patients should be investigated as adults considering the need to reduce, as much as possible, the exposure to ionizing radiation.

#### Kidney

- *The choice of diagnostic method upon admission depends on the hemodynamic status of the patient. (GoR 1A)*
- *E-FAST is effective and rapid to detect intra-abdominal free fluid. (GoR 1A)*
- *E-FAST has low sensitivity and specificity in kidney trauma. (GoR 1B)*
- *Contrast-enhanced CT scan associated with delayed urographic phase is the gold standard in hemodynamic stable or stabilized adults after blunt of penetrating trauma and in severely injured children when kidney or urinary tract injury is suspected. (GoR 1A)*
- *In blunt trauma, contrast-enhanced CT scan associated with delayed urographic phase must be performed in cases of macro- or micro-hematuria with hypotension and after high-energy deceleration trauma regardless of the presence of hematuria. (GoR 2B)*
- *In penetrating trauma, contrast-enhanced CT scan associated with delayed urographic phase is indicated in all hemodynamic stable or stabilized patients. (GoR 1B)*
- *Pediatric patients with high energy/penetrating/decelerating trauma and/or in cases of drop in hematocrit associated with any degree of hematuria should undergo contrast-enhanced CT-scan with delayed urographic phase. (GoR 2A)*
- *Ultrasound, contrast-enhanced US and eco-Doppler (E-FAST excluded) are generally not recommended as diagnostic tools during the initial evaluation of adult patients with high-energy trauma when multiple injuries and/or injury to the urinary tract and collecting system are suspected. (GoR 1C)*
- *Ultrasound, contrast-enhanced US, and eco-Doppler can be used in pregnant women and in the pediatric population as an alternative to CT scan in the presence of hemodynamic stability during the immediate assessment and in follow-up evaluations. (GoR 1C)*
- *In children with mild symptoms, minimal clinical findings, hematuria <50 RBCs/HPF and no other indications of CT-scanning, ultrasound and/or contrast-enhanced US and/or eco-doppler associated to blood test may be adopted for the initial evaluation. (GoR 2A)*
- *Intravenous urography may be useful in unstable patients during surgery when a kidney injury is found intraoperatively or when CT-scanning is not available and a urinary tract injury is suspected. (GoR 2C)*

**Extended-focused abdominal sonography for trauma (E-FAST)**, **Ultrasonography, and Doppler-US (DUS)** are useful and reliable noninvasive methods in trauma in general [69–71], however for the assessment of the kidney, due to anatomical reasons, these modalities may underestimate injuries (up to 30%) with a sensitivity and specificity of 22–67% and 96–100%, respectively [5, 14, 16, 17, 72–76]. In particular, vascular injuries are difficult to detect even using DUS [73].

In **children,** these are the methods of choice during follow-up excluding patients requiring CT-scan examination for other associated injuries [27, 77]. Usually, US/DUS can be safely used in the first 36–48 h reserving CT for selected cases or in cases of anomalies seen on US/DUS studies [22, 26, 77].

**Contrast-enhanced US (CEUS)** is not widely used [74, 78, 79]. Recent studies evaluated its use in abdominal trauma in the pediatric population and in fertile women as these methods seem to be effective in identifying extravasation, thrombosis, pseudoaneurysms (PSA), and post-traumautic arteriovenous fistulas [15, 80–86]. Contrast-enhanced US is thought to increase the accuracy of the E-FAST (above 80%) in stable patients in whom renal injuries are suspected but with a negative FAST or in the presence of hematuria, severe abdominal trauma, fertile women, pediatric patients, and in immediate or middle/long-term follow-up [72, 74, 76, 79–81, 86–89]. Some authors suggest using CEUS in patients with moderate and severe injuries to identify bleeding and inject a hemostatic agent percutaneously [80, 87]. Innovative US techniques with real-time 3D-enhanced imaging are promising in detecting ongoing hemorrhage [16, 90]. CEUS is not recommended in cases of suspicion of injury to the urinary tract and collecting system [85]. In these cases, contrast-enhanced CT-scan with late urographic phase is recommended.

**CT scan** with intravenous contrast is considered the gold standard in blunt and penetrating trauma [14, 15, 17, 75, 91–95]. In renal and urogenital trauma, the arterial and venous phases (20–30 s and 70–80 s of delay in acquiring the images, respectively) allow identification of almost all injuries and the addition of a 5-min delayed phase (excretory phase) permits the identification of urinary extravasation [5, 13, 14, 16, 75, 96–99]. This delayed phase should be added selectively in case of suspicion of urogenital injuries. CT-scanning should always be considered in patients with associated severe brain injury and in any major injuries for the high probability of occurrence of associated injuries [100]. Three-dimensional CT reconstructions help in injury classification [95, 101, 102]. the CT cystogram is a useful and viable tool and more accurate than plain X-ray cystography [14].

CT-scanning allows the identification of patients with high-risk criteria for NOM failure such as contrast blush, perirenal hematoma > than 3.5 cm, medial laceration with significant medial urinary extravasation (posteromedial blush/medial renal laceration) and lack of contrast in the ureter, suggesting a complete ureteropelvic junction disruption. The association of moderate or severe injuries and at least 2 of these criteria lead to a high rate to NOM failure [16, 103].

Routinely repeating CT scanning after trauma or in the follow-up phase is not recommended. A repeat CT-scan should be reserved for those cases with evident or suspected complications or significant clinical changes in moderate and severe injuries [15, 17, 75, 104, 105].

In the pediatric population, CT scanning to evaluate kidney injuries remains the gold standard in hemodynamic stable or stabilized patients with penetrating trauma or in cases where abdominal injuries are suspected independently to the grade of hematuria, when urogenital injury is suspected [10, 13, 20, 21, 24, 26, 33, 34, 106, 107]. In general, hospital CT-scan protocols should be adjusted to the ALARA (as low as reasonable achievable) principles of exposure to ionizing radiation [24, 106].

**Retrograde urethrography, excretory urethrography, and intravenous urography**

**Intravenous urography (IVU)** has been almost completely replaced by CT-scanning. However, it should be used in kidney injuries discovered during surgery in unstable patients, before opening the retroperitoneal hematoma. IVU can also be used when CT is not available or in low resource settings [3, 10, 13, 14, 18, 23, 36, 105, 108]. However, IVU is frequently used by urologists, more than by trauma surgeons [10]. The IVU false negative rate ranges between 37 and 75% [66].

The use of **excretory urethrography** has been reduced during the last decade in favor of contrast-enhanced CT-scan with delayed (excretory) phase [17]. However, in perineal trauma and/or in trauma in which pielo-uretral injuries, ureteral injuries, and bladder injuries are suspected, it might be useful [5, 109]. Another affordable tool to evaluate the urethra, especially in the operating room or in low resource settings is **retrograde urethrography**. Documenting a normal urethra prior to urinary catheterization in cases with a high level of suspicion for urethral lesions is advisable.

**Magnetic resonance image**

MRI can be used to diagnose renal trauma in fertile/pregnant women, in pediatric patients, in cases of iodine allergy, in some cases when CT images are equivocal, and in the follow-up phase of urinary tract injuries [15, 85, 110–112].

***Ureter***

- *Injury to the ureter should be suspected in high-energy blunt trauma, particularly in deceleration injuries with multi-system involvement and in all penetrating abdominal trauma. (GoR 1C).*
- *Intravenous contrast-enhanced CT-scan with delayed phase should be performed in hemodynamically stable or stabilized patients if ureteral injury is suspected (GoR 1C)*
- *Direct inspection of the ureter should be always performed during emergency laparotomy in patients with suspected ureteral injury. (GoR 1C)*

Perirenal stranding or hematomas, extravasation of contrast into the perirenal space, low-density retroperitoneal fluid around the genitourinary elements at imaging are indicative of ureteral injuries [49, 113]. Macro- and microscopic hematuria [114, 115] are not reliable signs of ureteral injury because its absence occurs in up to 25% of cases. A delay in the diagnosis may have a negative impact on outcomes [41, 113]. Ultrasound plays no role in the diagnosis of ureteral injury [49]. At Ct-scan with delayed phase peri-ureteral hematoma, partial or complete obstruction of the lumen, mild distension of the ureter, hydronephrosis, delayed pyelogram, and the lack of contrast in the ureter distal to the injury, are all signs suggestive of ureteral injury [50]. Urinary ascites or urinoma are considered subacute/chronic findings [44, 48]. A 10-minute delayed-phase CT-scan represents a valid diagnostic tool in the diagnosis of ureteral and ureteropelvic injuries [41, 113].

In case of unclear CT-scan results, an ascending urography represents the method of choice. IVU represents an unreliable test (false negatives up to 60%) [44, 114].

In case of emergency laparotomy, direct inspection of the ureter is indicated and it can be associated with the use of renally excreted intravenous dye (i.e., indigo carmine or methylene blue) [50]. Single-shot IVU may be indicated intraoperatively.

#### Bladder

- *Retrograde cystography (conventional radiography or CT-scan) represents the diagnostic procedure of choice in bladder injuries. (GoR 1C)*
- *Retrograde cystography should be always performed in hemodynamically stable or stabilized patients with suspected bladder injury. (GoR 1C)*
- *Intravenous contrast-enhanced CT-scan with delayed phase is less sensitive and specific than retrograde cystography in detecting bladder injuries. (GoR 1B)*
- *In pelvic bleeding amenable to angioembolization associated with suspected bladder injuries, cystography should be postponed until the completion of the angiographic procedure to avoid affecting the accuracy of angiography. (GoR 2A)*
- *Direct inspection of the intraperitoneal bladder, whenever feasible, should always be performed during emergency laparotomy in patients with suspected bladder injury. Methylene blue or indigo carmine could be useful in intraoperative investigation. (GoR 1C)*

In the presence of a pelvic fracture, macro-hematuria is associated with a bladder injury in almost one-third of cases and therefore represents an absolute indication for imaging of the bladder [48, 50]. However, micro-hematuria is not an indication for mandatory radiologic evaluation. Cystography should always be considered if other indicators of bladder injury are present such as low urine output, abdominal distension, inability to void, suprapubic tenderness, uremia or elevated creatinine level and entrance/exit wounds in the lower abdomen, perineum, or buttocks [54].

Conventional or CT-scan cystography has similar sensitivity and specificity in identifying bladder injuries (for 95% and 100% respectively). Whenever possible CT-scan cystography would be preferred [41, 45, 48, 116–118].. If associated urethral injury is suspected, a retrograde urethrography should be obtained before bladder catheterization. Passive anterograde distension of the bladder with exclusive renal-excreted contrast by clamping of the urinary catheter during abdominopelvic CT is not an effective maneuver to diagnose bladder rupture due to the high false negative rate caused by the low intravesical urine pressure [41, 48, 49, 119]. A technical pitfall of conventional cystography is represented by the false negative results in case of injuries located in the posterior wall: the lateral view is in fact rarely feasible due to the extent of pelvic injuries. In case a bladder injury is suspected in the presence of a bleeding pelvic fracture possibly amenable to angiographic management, caution should be used as extravasated contrast in the pelvis may impair the accuracy of the angiography [49].

#### Urethra

- *Patients with post-traumatic urethral hemorrhage should be investigated for urethral injuries. (GoR 1C)*
- *During emergency laparotomy, if an urethral injury is suspected, it should be investigated directly whenever feasible. (GoR 2A)*
- *Retrograde urethrography and selective urethroscopy represent the modalities of choice to investigate traumatic urethral injuries. (GoR 1B)*
- *In the event of penile lesions, urethroscopy should be preferred to retrograde urethrography (GoR 2A)*

Patients with urethral trauma may present with blood at the external urethral meatus, suprapubic fullness, perineal laceration, scrotal hematoma, urinary retention, difficulty or inability to insert a urinary catheter, and superiorly displaced prostate on rectal examination [45, 50, 68, 120, 121].

If urethral injury is present or suspected, rectal and vaginal examination should be performed. Associated rectal injuries are present in up to 5% of cases [121, 122].

There are two diagnostic modalities: retrograde urethrography and flexible urethroscopy [12, 58, 68].

If urethral injury is suspected, retrograde urethrography is the procedure of choice and should be performed before attempting any other maneuvers on the genito-urinary system [45, 48, 66, 123, 124].

In case of hemodynamic instability, all the investigations on the urethra should be postponed and a urinary drainage, (i.e., suprapubic catheter) should be inserted. The placement of a urethral catheter should be postponed until urethrography is obtained.

Extravasation of contrast on retrograde urography indicates an urethral injury [45]. Pelvic MRI, although not indicated in the acute setting, represents a valuable tool for anatomic definition of the injury during the post-traumatic period [48].

A distinction between incomplete and complete urethral lesions is difficult; in general, incomplete lesions identified on retrograde urography are often characterized by extravasation of contrast which also fills the bladder, whereas extravasation of contrast is not accompanied by bladder filling in complete lesions [120].

In case of associated penile injuries and in women due to short urethra, urethroscopy is recommended over retrograde urethrography [67, 124–127].

### Management

#### Kidney injuries 

**Non-operative management**

- *NOM should be the treatment of choice for all hemodynamical stable or stabilized minor (AAST I-II), moderate (AAST III) and severe (AAST IV-V) lesions. (GoR 1B)*
- *Only in selected settings, with immediate availability of operating room, surgeons and adequate resuscitation, immediate access to blood, blood products and to high dependency/intensive care environment, and without other reasons for surgical exploration, NOM may be considered even in hemodynamically transient responder patients. (GoR 2C)*
- *In deciding for NOM in hemodynamically stable or stabilized patients, accurate classification of the degree of injury and associated injuries with CT-scan with intravenous contrast and delayed urographic phases is mandatory. (GoR 2A)*
- *NOM in penetrating lateral kidney injuries is feasible and effective but accurate patient selection is crucial even in the absence of other indications for laparotomy. In particular, cases without violation of the peritoneal cavity are more suitable for NOM. (GoR 2A)*
- *Isolated urinary extravasation, in itself, is not an absolute contra-indication to NOM in absence of other indications for laparotomy. (GoR 1B)*
- *In low resource settings, NOM could be considered in hemodynamically stable patients without evidence of associated injuries, with negative serial physical examinations and negative first level imaging and blood tests. (GoR 2C)*

No specific recommendations exist for NOM in blunt and penetrating kidney and urogenital tract injuries in children that are different than those used for adults. Therefore, pediatric patients should be treated as adult patients keeping into account the rule that being less invasive is better.

NOM in severe injuries should be considered only in those settings where close clinical observation and hemodynamic monitoring in a high dependency/intensive care environment are possible, including serial clinical examination and laboratory tests, immediate access to diagnostics, interventional radiology and surgery, and immediately available access to blood and blood products. Alternatively, NOM may be used selectively if a system for immediate transfer to a higher level of care facility exists. NOM should be considered a step-wise approach starting with conservative management, followed by the use of minimally invasive (endoscopic or angiographic) techniques [92, 116, 128].. NOM lead to a higher renal preservation rate, a shorter hospital stay and a comparable complication rate to OM [128–141]. In hemodynamically stable or stabilized patients a CT scan with contrast together with delayed images is the gold standard to select patients for NOM [1, 17, 43, 92, 108, 116, 118, 130, 131, 133, 135, 138, 139, 141–160]. Incomplete staging is a relative indication to surgical exploration [133, 156, 159–161]. Non-resolving urinomas are common complications of NOM requiring ureteric stenting or percutaneous drainage [116, 128, 145, 147, 156, 158, 161]; perirenal hematoma and renal fragmentation are not absolute indications for acute OM [108, 146, 161].

Renal pelvis injury does not contraindicate NOM; however, it may request acute or delayed, endoscopic or open repair [17, 116, 147–149], particularly when complete avulsion of the ureteropelvic junction is observed.

Angioembolization of severe injuries allows continuation of NOM if after the procedure patients recovered from a hemodynamic point of view, and when no other indications for laparotomy exists [1, 17, 43, 116, 118, 135, 147, 150, 154, 161, 162]. In fact, In experienced centers with hybrid operating rooms, NOM may be attempted even in cases with a transient response to fluid resuscitation [1, 116] provided that all resources necessary for immediate operative intervention exist.

Isolated penetrating injuries to the kidney are rare; they are often associated with severe injuries, multiorgan involvement, and hemodynamic instability [1, 43, 92, 137, 145, 158, 159, 163]. However, NOM may be an appropriate first-line management option in hemodynamically stable patients without other indications for open surgical exploration (peritonitis, failed embolization, persistent bleeding, expanding or pulsatile hematoma, pielo-ureteral lesions) following penetrating trauma [1, 43, 92, 116, 128, 129, 135, 136, 138, 143, 144, 146–149, 154, 156, 159, 161, 164, 165]. As for blunt trauma, in deciding the applicability of NOM, institutional factors must be considered [92, 116, 128, 130, 132, 135, 141, 143, 147, 150, 160, 161]. Moreover, a multidisciplinary approach is needed [132, 141, 143, 144, 161]. It has been demonstrated that the degree of expertise of the trauma center plays a role in the successful rate of NOM [130, 132, 136, 161, 166]. Success rate of NOM is approximately 50% in stab wounds and 40% in gunshot wounds [1, 43, 137, 146, 150, 160].

Hemodynamically unstable patients with renal trauma not responsive to fluid resuscitation should undergo OM [1, 92, 108, 116, 142, 154–156, 158, 159].

No data exist regarding the best management strategy in low resource settings, although it seems rational to use OM in those circumstances. Low resource settings, in a limited sense, could be considered similar to military settings where lack of well-equipped hospital facilities, increased distance from trauma centers, and long transport time to definitive care facilities are the norm [167].

Other imaging modalities such as intravenous pyelography (less effective than CT in diagnosing significant renal injury) [43, 108, 116, 139, 154, 155, 158, 164], plain radiography [159], ultrasound (can lead to some significant false negative) [116, 139, 155, 157, 159] should be used to assess hemodynamically stable patients when CT scanning is not available.

Serial physical examination is reliable in detecting significant injuries after penetrating trauma to the abdomen [130, 164, 166] if performed by experienced clinicians and preferably by the same team.

**Operative management**

- *Hemodynamically unstable and non-responder (WSES IV) patients should undergo OM. (GoR 2A)*
- *Resuscitative Endovascular Balloon Occlusion of the Aorta (i.e., REBOA) may be used in hemodynamically unstable patients as a bridge to other more definitive procedures for hemorrhage control. (GoR 2B)*
- *In cases of severe renal vascular injuries without self-limiting bleeding, OM is indicated. (GoR 1C)*
- *The presence of non-viable tissue (devascularized kidney) is not an indication to OM in the acute setting in the absence of other indications for laparotomy. (GoR 2A)*
- *Hemodynamic stable or stabilized patients having damage to the renal pelvis not amenable to endoscopic/percutaneous techniques/stent should be considered for delayed OM in absence of other indications for immediate laparotomy. (GoR 2B)*

Uncontrollable life-threatening hemorrhage with avulsion of the renal pedicle and pulsating and/or expanding retroperitoneal hematoma or renal vein lesion without self-limiting hemorrhage are indications for OM. Retroperitoneal hematoma discovered during laparotomy and not adequately studied requires exploration of the kidney if they are pulsatile or if they are the only cause of hemodynamic instability. Whenever possible, the appropriate intraoperative diagnostic study should be performed [10, 13, 15, 18, 75, 132, 136, 137, 168–179]. All penetrating injuries associated with a retroperitoneal hematoma, if not adequately studied, should be explored especially if entering the peritoneal cavity [15, 137]. A shattered kidney or avulsion of the pyelo-ureteral junction in a hemodynamically stable patient do not mandate urgent surgical intervention. Arterial injuries or severe parenchymal injuries often result in nephrectomy when discovered intraoperatively [168, 179]. The success rate of arterial repair is 25–35% [15, 18, 177]. Arterial repair should be attempted in cases of patients with only one kidney or in those with bilateral renal injuries. Urine extravasation is not by itself an indication for OM in the acute setting [18, 169, 180].

Some cases of renal injury result in significant devascularization of the organ which results in a significant renin-angiotensin-aldosterone cascade response. These patients may complain of flank pain and have unrelenting persistent hypertension not responsive to anti-hypertensives. In these rare instances, and when a contralateral kidney is functional, nephrectomy may be the only option if all other management strategies fail.

**Angiography and angioembolization**

- *Angiography with eventual super-selective angioembolization is a safe and effective procedure; it may be indicated in hemodynamically stable or stabilized patients with arterial contrast extravasation, pseudoaneurysms, arteriovenous fistula, and non-self-limiting gross hematuria. (GoR 1C)*
- *Angioembolization should be performed as selectively as possible. (GoR 1C)*
- *Blind-angioembolization is not indicated in hemodynamically stable or stabilized patients with both kidneys when angiography is negative for active bleeding, regardless of arterial contrast extravasation on CT-scan. (GoR 1C)*
- *In hemodynamically stable or stabilized patients with severe renal trauma with main renal artery injury, dissection or occlusion, angioembolization and/or percutaneous revascularization with stent or stentgraft is indicated in specialized centers and in patients with limited warm ischemia time (< 240 min) (GoR 2C)*
- *Endovascular selective balloon occlusion of the renal artery could be utilized as a bridge to definitive hemostasis. This procedure requires direct visualization by fluoroscopy where the balloon is advanced over a selectively placed guidewire. (GoR 2B)*
- *In severe injury with main renal vein injury without self-limiting bleeding, angioembolization is not indicated. Patients should undergo surgical intervention. (GoR 1C)*
- *In hemodynamically stable or stabilized patients with solitary kidney and moderate (AAST III) or severe (AAST IV–V) renal trauma with arterial contrast extravasation on CT-scan, angiography with eventual super-selective angioembolization should be considered as the first choice. (GoR 1C)*
- *In hemodynamically stable or stabilized patients with active kidney bleeding at angiography and without other indications for surgical intervention, in case of failure of the initial angioembolization, a repeat angioembolization should be considered. (GoR 1C)*
- *In adults, only in selected setting (immediate availability of operating room, surgeon, adequate resuscitation, immediate access to blood and blood products and to high dependency / intensive care environment) and without other reasons for surgical exploration, angioembolization might be considered in selected hemodynamically transient responder patients. (GoR 2C)*
- *In children, angiography and eventual super-selective angioembolization should be the first choice even with active bleeding and labile hemodynamics, if there is immediate availability of angiographic suite, immediate access to surgery and to blood and blood products, and to high dependency / intensive care environment. (GoR 2C)*

Indications to angiography and eventual selective angioembolization include arterial contrast extravasation on CT-scan in hemodynamically stable or transient responder patients [170, 181–188], gross non-self-limiting hematuria [188, 189], arteriovenous fistula [181, 188], Pseudoaneurysm (PSA) [188, 190] extended perirenal hematoma [184, 186, 191, 192] and progressive decrease in hemoglobin concentration during NOM [185, 188]. Disrupted Gerota’s fascia associated with contrast extravasation is suggested to increase the need for AE [192]. The grade of parenchymal disruption seems not to be associated with AE need even if severe renal injuries are associated with a reduced rate of AE success [170, 183, 186, 193]. Almost 32% of blunt renal injuries with arterial contrast extravasation on CT-scan have negative angiography [182]; these cases can be successfully managed without AE [182]. Overall AE success rate in blunt renal trauma ranges from 63% to 100% [135, 162, 181, 185, 188, 189, 194–200]. In case of need for a repeat AE, the success rate is similar to those seen in initial AE, so re-interventions are justified when indicated by the clinical course [185]. Failure rates are linked to the experience of the centers [199]. AE seems to have better results in terms of renal function and ICU length of stay compared with nephrectomy, showing similar transfusion need and re-bleeding rates [200].

The anatomical damage to the kidney is associated with the need to repeat AE [193], but not with an overall AE failure [170]. Kidney devascularisation, initial hemodynamic instability, low hemoglobin concentration, the ISS, and associated injuries did not correlate with a higher rate of AE failure [170, 193]. Age and volume of blood products given in the first 24 h, the experience of the center, and penetrating trauma are associated with a higher risk of AE failure [193].

Renal AE has lower complication rates compared with surgery [162]. Renal dysfunction or renovascular hypertension directly linked to AE for renal injury is rare [162, 185, 186, 189, 197, 200–203].

Long-term follow-up showed good functional and morphological results in patients with single kidney [198]. Reported morbidity rate after AE is 25% [135, 189, 192] and includes accidental embolization of healthy arterial branches of vascularised territories, puncture-site bleeding, arterial dissection and thrombosis, contrast-induced nephropathy, post-embolization syndrome (i.e., back pain and fever), gross hematuria, renal abscess, coils migration, PSA and arteriovenous fistulae [162, 188, 189].

Shattered kidney without renal hilum avulsion could be treated with AE [185, 194], but the management of renal pedicle avulsion is still a matter of debate, with some reporting AE success rates of 80% but with the need of repeat angioembolization in almost all cases [170, 193, 204, 205], and others reporting a failure rate of 100% [188].

Renal venous pedicle avulsion becomes the only contraindication for NOM and AE and requires immediate surgery [181, 186].

Accumulating evidence exists regarding the successful use of AE even in patients with severe trauma with liable hemodynamic parameters provided that the environment is adequate and risk is not increased [170, 186, 194, 204, 205]. In general, one in five penetrating kidney injury patients initially treated with conservatively will need either surgical or angiographic mamagement [206]. Reported AE success rate after renal stab wounds with vascular injuries is 82-88% [203, 207]. Embolization should be performed as sub-selectively as possible to limit the associated parenchymal infarction [208]. Agents used for AE can induce either temporary or permanent arterial occlusion. The chosen embolic agents depend on the type of vascular injury (direct bleeding, PSA, arteriovenous fistula), but the majority of procedures are performed using coils with or without gelfoam [162].

Results of kidney artery surgical revascularization are poor, with long-term kidney function preservation rate of less than 25% [209, 210]. Conservative management of main renal artery occlusion leads to a high rate of severe hypertension, requiring subsequent nephrectomy. Percutaneous revascularization with stents showed better outcomes on renal function than surgical treatment [209, 210]. However, it must be pointed out that warm ischemia time longer than 60 min leads to significant exponential losses in kidney function [211, 212]. The placement of a peripheral stent graft may be considered for hemostasis allowing perfusion of the renal artery distal to the injury site. Selective balloon occlusion can be considered as a temporary bleeding control maneuver prior to laparotomy however fluoroscopy is required for positioning of the guidewire and balloon catheter. Selective renal artery balloon occlusion leads to less global ischemia compared with aortic balloon occlusion.

Present guidelines and WSES classification consider segmental vascular injuries (SVI) as moderate lesions due to the reduced risk of organ loss and minor risk for life loss. Moreover, they have been separated from collecting system lacerations (CSL) as the overall NOM successful rate is significantly lower in SVI when compared with CLS (43% vs. 98%) [173]. SVI may be successfully treated with AE [116, 207].

The reported success rate of AE in children with blunt renal trauma and contrast medium extravasation or PSA is 100% with a major morbidity rate of 0% [213–215].

Current indications for AE in children are not universally recognized and include moderate and severe injuries, active bleeding with contrast blush on CT-scan, ongoing hemodynamic instability and PSA [215–217] with the suggestion to proceed with NOM only in those environments allowing for it without any additional risk. AE in pediatrics fills a void between NOM in the hemodynamically stable children and OM in the highly unstable patient with severe renal injury [217].

#### Urinary tract injuries

***Ureter***

- *Contusions may require ureteral stenting when urine flow is impaired. (GoR 1C)*
- *Partial lesions of the ureter should be initially treated conservatively with the use of a stent, with or without a diverting nephrostomy in the absence of other indications for laparotomy. (GoR 1C)*
- *Partial and complete ureteral transections or avulsion not suitable for NOM may be treated with primary repair plus a double J stent or ureteral re-implant into the bladder in case of distal lesions (GoR 1C).*
- *Ureteral injuries should be repaired operatively when discovered during laparotomy or in cases where conservative management has failed (GoR 1C)*
- *Ureteral stenting should be attempted in cases of partial ureteral injuries diagnosed in a delayed fashion; if this approach fails, and/or in case of complete transection of the ureter, percutaneous nephrostomy with delayed surgical repair is indicated. (GoR 1C)*
- *In any ureteral repair, stent placement is strongly recommended. (GoR 1C)*

In the absence of other indications for laparotomy, the majority of low-grade ureteral injuries (contusion or partial transection) may be managed by observation and/or ureteral stenting [43, 115]. If stenting is unsuccessful, a nephrostomy tube should be placed [45]. If ureteral injuries are suspected during a laparotomy, direct visualization of the ureter is mandatory [43]. Whenever possible, ureteral injuries should be repaired. Otherwise, a damage control strategy should be preferred, with ligation of the damaged ureter and urinary diversion (temporary nephrostomy), followed by delayed repair [45, 50, 115]. In cases of complete transection of the ureter, surgical repair is indicated [43]. The two main options are primary uretero-ureterostomy or ureteral re-implant with bladder psoas hitch or a Boari flap [43, 50, 114, 115, 218]. The use of ureteral stents is recommended after all surgical repairs to reduce failures (leaks) and strictures [13, 42, 45, 50, 116, 127]. Distal injuries to the ureter (caudal to the iliac vessels) are usually treated by reimplantation of the ureter in the bladder (uretero-neocystostomy), as the traumatic insult may jeopardize the blood supply [42, 43, 45, 50, 218]. In cases of delayed diagnosis of incomplete ureteral injuries or delayed presentation, an attempt of ureteral stent placement should be done; however, retrograde stenting is often unsuccessful. In these cases, delayed surgical repair should be considered [219].

***Bladder***

- *Bladder contusion requires no specific treatment and might be observed clinically. (GoR 1C)*
- *Intraperitoneal bladder rupture should be managed by surgical exploration and primary repair (GoR 1B)*
- *Laparoscopy might be considered in repairing isolated intraperitoneal injuries in case of hemodynamic stability and no other indications for laparotomy. (GoR 2B)*
- *In case of severe intraperitoneal bladder rupture, during damage control procedures, urinary diversion via bladder and perivesical drainage or external ureteral stenting may be used. (GoR 1C)*
- *Uncomplicated blunt or penetrating extraperitoneal bladder injuries may be managed non-operatively, with urinary drainage via a urethral or suprapubic catheter in the absence of other indications for laparotomy. (GoR 1C)*
- *Complex extra-peritoneal bladder ruptures—i.e., bladder neck injuries, lesions associated with pelvic ring fracture and/or vaginal or rectal injuries—should be explored and repaired. (GoR 1C)*
- *Surgical repair of extraperitoneal bladder rupture should be considered during laparotomy for other indications and during surgical exploration of the prevesical space for orthopedic fixations. (GoR 1C)*
- *In adult patients, urinary drainage with urethral catheter (without suprapubic catheter) after surgical management of bladder injuries is mandatory (GoR 1B); for pediatric patients, suprapubic cystostomy is recommended (GoR 2C)*

In cases of hemodynamic instability, urethral or suprapubic catheter may be inserted as a temporary measure and the repair of the bladder injury may be postponed [45].

All penetrating bladder injuries and Intraperitoneal bladder rupture (IBR) generally require surgical exploration and primary repair [41, 45, 53]. Laparoscopic repair of isolated IBR is a viable option [220]. Open surgical repair of bladder injuries is in a double-layer fashion using monofilament absorbable suture [54]. Single-layer repair is common during laparoscopic approach [12, 45, 54, 221, 222].

Uncomplicated blunt or penetrating EBR, in the absence of other indications for laparotomy, may be managed conservatively, with clinical observation, antibiotic prophylaxis and the insertion of a urethral catheter or a suprapubic percutaneous cystostomy, in case of a concomitant urethral injury [45]. Injury healing happens within 10 days in more than 85% of cases [53]. Surgical repair of EBR is indicated in complex injuries as bladder neck injuries or injuries associated with pelvic fractures requiring internal fixation and rectal or vaginal injuries [41, 50]. Furthermore, surgical repair of EBR may be considered in case of non-resolution of urine extravasation 4 weeks after the traumatic event [45].

Gunshot injuries of the bladder are commonly associated to rectal injuries, which prompt fecal diversion. Commonly, these injuries are through-and-through (entry/exit site) requiring careful and complete pelvic inspection [222].

Urethral catheterization whenever possible has the same efficacy of suprapubic cystostomy; therefore routine placement of a suprapubic tube is no longer recommended [45, 223, 224]. Suprapubic catheterization may be reserved for cases with associated perineal injuries. Suprapubic drainage is recommended in children after the surgical repair of bladder rupture [225].

***Urethra***

- *Urinary drainage should be obtained as soon as possible in case of traumatic urethral injury. (GoR 1C)*
- *Blunt anterior urethral injuries should be initially managed conservatively with urinary drainage (via urethral or suprapubic catheter); endoscopic treatment with realignment should be attempted before surgery. Delayed surgical repair should be considered in case of failure of conservative treatment after endoscopic approach. (GoR 1C)*
- *Partial blunt injuries of the posterior urethra may be initially managed conservatively with urinary drainage (via urethral or suprapubic catheter) and endoscopic realignment; definitive surgical management should be delayed for 14 days if no other indications for laparotomy exist. (GoR 1C)*
- *Injuries of the posterior urethra in cases of hemodynamic instability should be approached by immediate urinary drainage and delayed treatment. (GoR 1C)*
- *Conservative treatment of penetrating urethral injuries is generally not recommended. (GoR 1C)*
- *Penetrating injuries of anterior urethra should be treated with immediate direct surgical repair if the clinical conditions allow and if an experienced surgeon is available; otherwise, urinary drainage should be performed and delayed treatment planned. (GoR 1C)*
- *Penetrating injuries of the posterior urethra should be treated with primary repair only if the clinical conditions allow. Otherwise, urinary drainage and delayed urethroplasty are recommended. (GoR 1C)*
- *When posterior urethral injury is associated with complex pelvic fracture, definitive surgical treatment with urethroplasty should be performed after the healing of pelvic ring injury. (GoR 1C)*

*·*

Bladder drainage should be obtained soon and as safe and technically feasible. In case of contrast extravasation on urethrogram, a suprapubic catheter should be considered [57, 226].

The treatment of choice in case of penetrating urethral injuries is surgical exploration and repair [227, 228]. Posterior urethral blunt injuries and selected penetrating partial injuries, in the absence of other indications for laparotomy, may be treated initially by NOM with the insertion of a suprapubic cystostomy or urethral catheter, as primary open realignment and primary open anastomosis are associated with high rates of stricture, urinary incontinence, and impotence [45, 50, 66, 123, 229].

However, the insertion of a suprapubic catheter may be difficult due to hematoma or to poor bladder filling in case of shock; an experienced provider may attempt once a careful urethral catheter placement [58, 60, 61, 67, 120, 125, 126, 226]. However, if any resistance is encountered, a suprapubic catheter should be placed under direct visualization or with ultrasound guidance [120].

In case of anterior urethral blunt trauma, the initial treatment of choice is conservative with urinary drainage (by suprapubic or urethral catheter placement) and delayed treatment after an accurate evaluation of the extent of the injury. A trial of endoscopic realignment should be undertaken. In case of failure, surgery is recommended with urethroplasty [67, 230]. Selected cases of incomplete penetrating injuries of the anterior urethra may be managed with trans-urethral catheter placement.

Urethrography should be performed every two weeks until complete healing [122].

Unless other life-threatening injuries are present, uncomplicated penetrating lesions of the anterior urethra are best managed with prompt direct surgical repair [124]. Cases in which damage control procedures are needed or in which anastomotic urethroplasty is not feasible due to a large anatomic defect (typically lesions > 2–3 cm in the bulbar urethra and > 1.5 cm in the penile urethra), marsupialisation of the urethra, temporary suprapubic urinary catheter placement and delayed anatomic reconstruction with graft or flap (interval urethroplasty at > 3 months) are indicated [45].

In blunt posterior urethral injuries, initial conservative treatment is recommended with planned delayed surgical treatment, allowing multidisciplinary management involving experienced surgeons and urologists [45].

In case of hemodynamically stable patients with complete lesions of the posterior urethra without other life-threatening injuries, immediate endoscopic realignment is preferred over immediate urethroplasty. Endoscopic realignment is associated with improved outcomes [67, 229, 231, 232]. Therefore, immediate urethroplasty is not routinely recommended. When endoscopic realignment is unsuccessful, urinary drainage with suprapubic catheter placement and delayed urethroplasty are indicated [123, 229], preferably within 14 days from the injury. In case of associated pelvic fractures, definitive surgery should be postponed until after the healing of pelvic ring injuries [50, 126, 222, 231, 233, 234].

The management of penetrating injuries to the posterior urethra depends on the presence and severity of associated injuries. In case of life-threatening associated injuries and Damage Control approach, urinary diversion and delayed urethroplasty is advised [64, 127].In hemodynamic stable patients, without associated severe injuries, immediate retropubic exploration and primary repair of the injury is recommended [64, 126].

**Follow-up:**

- *Follow-up imaging is not required for minor (AAST I-II) renal injuries managed non-operatively. (GoR 2B)*
- *In moderate (AAST III) and severe (AAST IV-V) renal injuries, the need for follow-up imaging is driven by the patients’ clinical conditions. (GoR 2B)*
- *In severe injuries (AAST IV-V), contrast-enhanced CT scan with excretory phase (in cases with possible or documented urinary extravasation) or ultrasound and contrast-enhanced US are suggested within the first 48 h after trauma in adult patients and in delayed follow-up. (GoR 2A)*
- *Follow-up imaging in pediatric patients should be limited to moderate (AAST III) and severe (AAST IV-V) injuries. (GoR 2B)*
- *In pediatric patients, ultrasound and contrast-enhanced US should be the first choice in the early and delayed follow-up phases. If cross-sectional imaging is required, magnetic resonance should be preferred. (GoR 2B)*
- *CT-scan with delayed phase imaging is the method of choice for the follow-up of ureteral and bladder injuries. (GoR 2A)*
- *Ureteroscopy or urethrogram are the methods of choice for the follow-up of urethral injuries. (GoR 2A)*
- *Return to sport activities should be allowed only after microscopic hematuria is resolved. (GoR 2B)*

In general mild and moderate injuries have a very low complication rate [235–237]. Routine follow-up imaging may not be justified for mild injuries [236–240]. In severe injuries, CT scan with delayed excretory phase is recommended within the first 48 h after admission as urinary leak may be missed on the initial CT scan in 0.2% of all cases and in 1% of high-grade renal injuries [105]..

Moderate injuries without urine extravasation would require follow-up imaging only in case of worsening of patient status [17, 236, 239, 241, 242].

The risk of secondary hemorrhage deserves particular mention. Secondary hemorrhage is usually caused by rupture of a PSA or arteriovenous fistula, which occurs in up to 25% of moderate/severe injuries [151, 243] within 2 weeks of the injury [151, 207, 243]. Hematuria is the most common sign suggesting these complications [151]. It is an indication to perform contrast-enhanced CT scan or DUS or CEUS, according to the availability of the tests in the hospital. These three techniques showed to be similar in reliability regarding the detection of these complications [77, 151].

No definitive evidence exists with regard to timing of return to normal activity after renal trauma. In general, bed rest or reduced activity is recommended until gross hematuria is resolved [146, 237, 244].

Return to sport activities after a minor or moderate renal injury may occur within 2 to 6 weeks from the injury while severe injuries may require longer periods (6 to 12 months) [245, 246]. As a general rule, sports activities should be avoided until microscopic hematuria is resolved [245, 246].

Limited low-grade evidence is available with regard to the best follow-up strategy in pediatric patients with renal trauma. US or CEUS may be considered the method of choice in moderate and severe renal injuries, even if initially evaluated by CT-scan [247]. If US or CEUS imaging is inconclusive MRI, if available, should be performed.

There is no sufficient evidence regarding the relationship between renal injury severity and the rate and timing of healing or incidence of renal dysfunction [247–249]. Low-grade kidney injuries have a very low rate of late complication in pediatric patients; therefore, scheduled imaging follow-up in the potential complications is not indicated [247, 250]. The reported incidence of renal trauma-induced hypertension is 0–6.6% [244, 251–254], but in general, all those who are normotensive in the immediate post-trauma period usually do not develop signs of hypertension during follow-up [251].

## Conclusions

The management of kidney and urogenital trauma is multidisciplinary. When feasible, non-operative management should always be considered as the first option. For this reason, the anatomy of the injury, its physiological effects, and the associated injuries should always be considered to define the best treatment strategy.

## Data Availability

Not applicable.

## Abbreviations

AAST: American Association for Surgery for Trauma
AG/AE: Angiography/angioembolization
ALARA: As low as reasonable achievable
BE: Base excess
CSL: Collecting system lacerations
CBR: Combined bladder rupture
CT: Computed tomography
CEUS: Contrast-enhanced ultrasound
DUS: Doppler-US
EVTM: Endovascular trauma and bleeding management
E-FAST: Extended-focused abdominal sonography for trauma
EBR: Extra-peritoneal bladder rupture
fNOM: Failure of NOM
GCS: Glasgow Coma Scale
HPF: High-power field
IBR: Intra-peritoneal bladder rupture
ISS: Injury severity score
IVU: Intravenous urography
LE: Level of evidence
MRI: Magnetic resonance image
MTP: Massive transfusion protocols
NOM: Non-operative management
OIS: Organ injury scale
OM: Operative management
PFUI: Pelvic fracture urethral injury
PSA: Pseudoaneurysm
RBCs: Red blood cells
REBOA: Resuscitative endovascular balloon occlusion of the aorta
SVI: Segmental vascular injuries
US: Ultrasound
WSES: World Society of Emergency Surgery
